# MoO_3−*x*_-deposited TiO_2_ nanotubes for stable and high-capacitance supercapacitor electrodes†

**DOI:** 10.1039/c8ra02744g

**Published:** 2018-06-13

**Authors:** Shupei Sun, Yu Sun, Jiang Wen, Bo Zhang, Xiaoming Liao, Guangfu Yin, Zhongbing Huang, Ximing Pu

**Affiliations:** College of Materials Science and Engineering, Sichuan University Chengdu Sichuan 610065 China sherman_xm@163.com +86 28 85413003

## Abstract

Here we report the supercapacitive properties of a novel MoO_3−*x*_/TiO_2_ nanotube composite prepared by a facile galvanostatic deposition technique and subsequently thermal treatment in an argon atmosphere between 350 °C and 550 °C. X-ray diffraction and X-ray photoelectron spectroscopy confirm the existence of MoO_3−*x*_. The MoO_3−*x*_/TiO_2_ electrode prepared at 550 °C exhibits a high specific capacitance of 23.69 mF cm^−2^ at a scan rate of 10 mV s^−1^ and good cycling stability with capacitance retention of 86.6% after 1000 cycles in 1 M Na_2_SO_4_ aqueous solution. Our study reveals a feasible method for the fabrication of TiO_2_ nanotubes modified with electroactive MoO_3−*x*_ as high-performance electrode materials for supercapacitors.

## Abstract 2

Here we report the supercapacitive properties of a novel MoO_3−*x*_/TiO_2_ nanotube composite prepared by a facile galvanostatic deposition technique and subsequently thermal treatment in an argon atmosphere between 350 °C and 550 °C.

## Graphical abstract

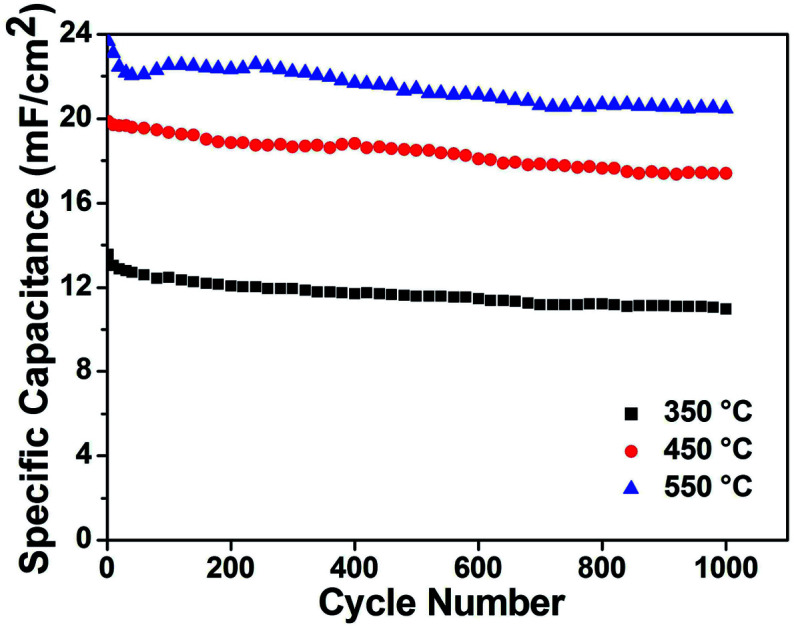

## 1. Introduction

In the past decade, various nanostructured materials (such as nanotubes,^1,2^ nanorods,^3,4^ nanowires,^5,6^ and nanofibers^7^) have been widely investigated because they possess ordered geometrical structure. In particular, TiO_2_ nanotubes (TNTs) with a well-defined open-top and uninterrupted nanotube structure have received tremendous attention in various applications including photocatalytic degradation of environmental pollutants,^8^ solar cells,^9^ energy storage devices,^10^ drug delivery^11^ and sensors.^12^ Supercapacitors are one of the most promising electrochemical energy-storage systems and have attracted tremendous attention.^13–17^ Recently, self-assembled titania nanotube arrays have received immense interest for use in supercapacitors because of their capability to offer large surface area for electrochemical reactions and unique electron transport pathways compared with non-oriented structures. Additionally, the electroactive materials can directly grow on the surface of TNTs, avoiding the use of a polymer binder and conductive additives.

However, TiO_2_ is known as an intrinsic n-type semiconductor with a band gap of approximately 3.2 eV (for anatase phase) and 3.1 eV (for rutile phase).^18^ Pristine TNTs only contribute a very low capacitance (0.047–0.97 mF cm^−2^).^19–22^ Therefore, TiO_2_ nanotubes need to be modified urgently in order to meet the demands for supercapacitors with high electrochemical performance. A promising method to further enhance the capacitance of TNTs is to incorporate with some transition metal oxides (NiO,^23^ MnO_2_ (ref. 24 and 25) and MoO_3_ (ref. 26) *etc.*). The composite electrode can utilize the positive features of individual materials and may provide a combined effect, such as large capacitance and good cycling performance.^27^

Molybdenum oxides have enabled a wide range of applications in gas sensing,^28^ catalysts,^29^ electrochromic devices^1^ and energy storage^30,31^ due to their low cost, resource abundance and nontoxicity. In particular, a layered molybdenum trioxide (α-MoO_3_) has been studied as an attractive electrode material for supercapacitor due to its high theoretical specific capacitance of 1111 mA h g^−1^.^32^ However, the poor electrical conductivity and large capacitance loss in initial cycles have limited its application as an electrode material.^26,33^ Annealing in inert or hydrogen atmosphere could effectively improve electrical conductivity of some transition metal oxides.^20,34^ Meduri *et al.* discovered that oxygen deficient MoO_3−*x*_ had fast intercalation and excellent electrochemical performance in lithium ion batteries.^6^ Law *et al.* reported that oxygen vacancies in MoO_3−*x*_ led to enhanced electrical conductivity.^35^ Dunn *et al.* showed that incorporation of oxygen vacancies in MoO_3−*x*_ could enhance pseudocapacitive charge storage properties.^36^ To the best of our knowledge, there have been a few studies on incorporating MoO_3−*x*_ with TiO_2_ nanotubes and thus exploring its supercapacitive behavior. Compared with other hybrid electrodes,^37–39^ the prepared electrodes can make full use of TiO_2_ nanotubes and MoO_3−*x*_ layer, thus achieving high electrochemical performance. Therefore, we fabricated MoO_3−*x*_/TiO_2_ nanotubes composite and extensively studied its application for supercapacitor.

In this work, a feasible approach was proposed to prepare MoO_3−*x*_/TiO_2_ nanotube hybrid by galvanostatic deposition in molybdenum salt solutions and subsequent thermal treatment in argon atmosphere. Various microstructure characterizations and electrochemical measurements were conducted to investigate the crystal phase, surface morphology, chemical composition and electrochemical properties of prepared samples. The electrochemical measurements reveal improved electrochemical performance of MoO_3−*x*_/TiO_2_ nanotubes composite with high specific capacitance as well as stable capacitance behavior.

## 2. Experimental procedure

### 2.1. Materials

The titanium sheet (TA2, purity > 99.6%, 10 × 10 × 1 mm^3^) was purchased from LuoKe titanium Ltd. Ethylene glycerol (C_2_H_6_O_2_, purity > 99.5%), ammonium fluoride (NH_4_F, purity > 96%), hexaammonium molybdate tetrahydrate ((NH_4_)_6_Mo_7_O_24_·4H_2_O, purity > 99.0%) and sodium sulfate anhydrous (Na_2_SO_4_, purity > 99.0%) were purchased from KeLong Chemical Ltd. Other chemical reagents used in these experiments were of analytical grade and were used directly without further purification. All aqueous solutions were prepared using deionized water.

### 2.2. Fabrication of MoO_3−*x*_/TiO_2_

The highly ordered TNTs were prepared as described elsewhere.^26^ In order to enhance adhesion of nanotubes to titanium substrate and obtain crystalline TNTs, the prepared nanotubes were annealed at 450 °C for 1 h in ambient atmosphere. Then electrochemical deposition was carried out with a three-electrode system composed of annealed TNTs as the working electrode, a Pt foil as the counter electrode and an Ag/AgCl as the reference electrode. Galvanostatic depositions were carried out at a current density of 0.5 mA cm^−2^ for 5 min in 0.05 M (NH_4_)_6_Mo_7_O_24_·4H_2_O aqueous solution. Afterwards, the as-deposited samples were annealed at 350 °C, 450 °C, and 550 °C in argon atmosphere for 1 h. In consideration of tubular structure collapses at higher annealing temperatures (*e.g.* 650 °C), we just studied capacitive behavior of deposited samples annealed at 350–550 °C. The sample annealed at 450 °C is designated as M-TNT. For comparison, TNTs just annealed at 450 °C for 1 h in ambient atmosphere are referred as TNT.

### 2.3. Materials characterization and electrochemical measurements

A Hitachi S-4800 scanning electron microscopy (SEM) and DX-2700 X-ray diffraction (XRD) were used to investigate the surface morphology and microstructure of the fabricated samples, respectively. Chemical composition and chemical states of deposited samples were characterized by X-ray photoelectron spectroscope (XPS, Escalab 250Xi, USA). Electrochemical measurements were performed in a 1 M Na_2_SO_4_ aqueous solution with an electrochemical workstation (CHI660E, China) in a 3-electrode setup consisting of the prepared sample as the working electrode, platinum foil as the counter-electrode, and saturated Ag/AgCl electrode as the reference electrode. Cyclic voltammetry (CV) tests were conducted over a voltage range from −0.9 to −0.2 V (*vs.* Ag/AgCl) at different scan rates (from 10 to 200 mV s^−1^). The galvanostatic charge–discharge (CD) testing was performed under different current densities (from 0.4 to 1 mA cm^−2^). Mott–Schottky plots were measured at a frequency of 1 kHz. Electrochemical impedance spectroscopy (EIS) measurements were conducted over a frequency range of 0.01 Hz to 100 kHz at an AC voltage amplitude of 5 mV. The cycling stability of the samples was tested by galvanostatic CD measurement performed up to 1000 cycles at a current density of 0.6 mA cm^−2^.

## 3. Results and discussion

### 3.1. Morphology and structure analyses

**Fig. 1:**
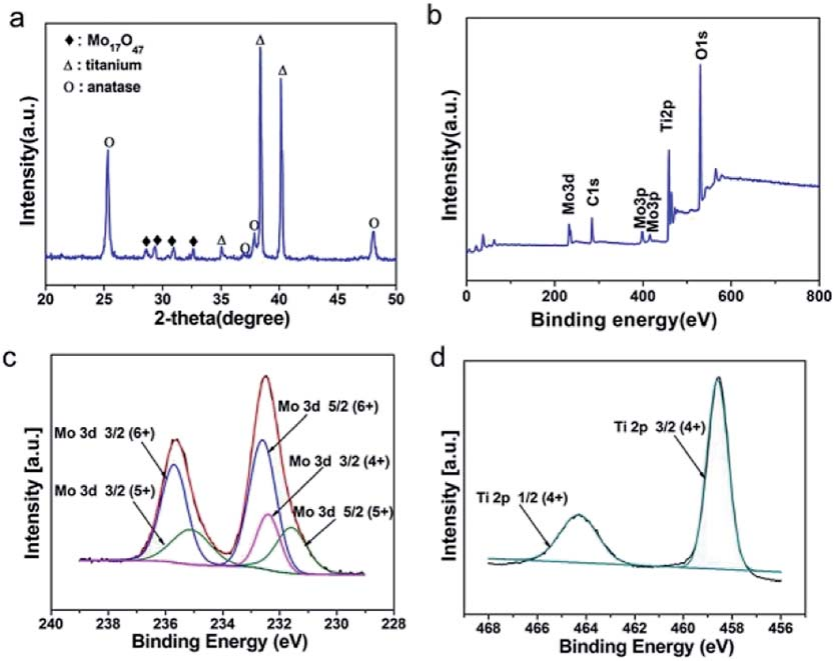
XRD pattern of M-TNT (a). XPS spectra of M-TNT: survey scan (b), Mo 3d (c) and Ti 2p (d) binding energy regions.

The crystalline phase of prepared M-TNT was characterized by XRD. As shown in Fig. 1a, the diffraction peaks located at ∼25.3° and ∼48.0° are the characteristic peaks of anatase TiO_2_ (JCPDF 21-1272) while peaks at ∼38.4° and ∼40.1° are assigned to Ti (from Ti substrate). Notably, the peaks with 2θ of ∼28.5°, ∼29.4°, ∼30.9° and ∼32.6° are indexed to an oxygen deficient Mo_17_O_47_ phase (JCPDF 71-0566).^6^ Due to the strong sharp diffraction peaks of titanium substrate, the diffraction peaks corresponding to MoO_3−*x*_ are suppressed.

**Fig. 2:**
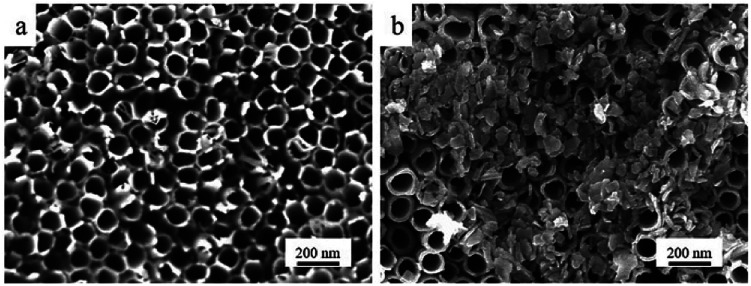
SEM images of TNT (a) and M-TNT (b).

Furthermore, XPS measurements were then carried out to determine valence states of M-TNT. In the survey spectrum (Fig. 1b), peaks of Mo and O are clearly observed, suggesting the presence of molybdenum oxides in the composite. Typical Mo 3d and Ti 2p spectra are shown in Fig. 1c and d, respectively. The Mo 3d5/2 peak at ∼231.6 and the Mo 3d3/2 peak at ∼235.1 eV correspond to Mo^5+^, and the Mo 3d3/2 peak at 232.4 eV corresponds to Mo^4+^, respectively.^40^ Our early research discovered that coating layer after electrochemical deposition in molybdenum salt solutions was just amorphous Mo^5+^ oxides and Mo^6+^ oxides.^26^ So, the formation of Mo^4+^ from M-TNT indicates that thermal treatment in argon atmosphere could lead to oxygen loss on the surface of deposition coating, resulting in the generation of oxygen deficiency and low oxidation states. This phenomenon has been previously observed in other reports.^41,42^ The peaks at ∼232.6 eV and ∼235.7 eV are indexed to 3d5/2 and 3d3/2 of Mo^6+^, respectively.^43,44^ Additionally, the Ti 2p3/2 peak at ∼458.6 eV and the Ti 2p1/2 peak at ∼464.3 eV correspond to Ti^4+^ (Fig. 2b),^45^ indicating the formation of TiO_2_. Therefore, in combination with above analysis we successfully prepared MoO_3−*x*_/TiO_2_ composite electrode material.

Fig. 2 shows the surface morphology of TNT and M-TNT. From Fig. 2a, the anodized TNTs with high-density, well-ordered and uniform characteristics can be seen. The whole surface of the nanotubes is bare without any loading or coating. As shown in Fig. 2b, MoO_3−*x*_ species are electrodeposited on the top of nanotubes to form a film-on-tube structure. Notably, some nanotubes still can be seen, which contribute to speedy permeation of electrolyte ions.

### 3.2. Electrochemical characterizations

**Fig. 3:**
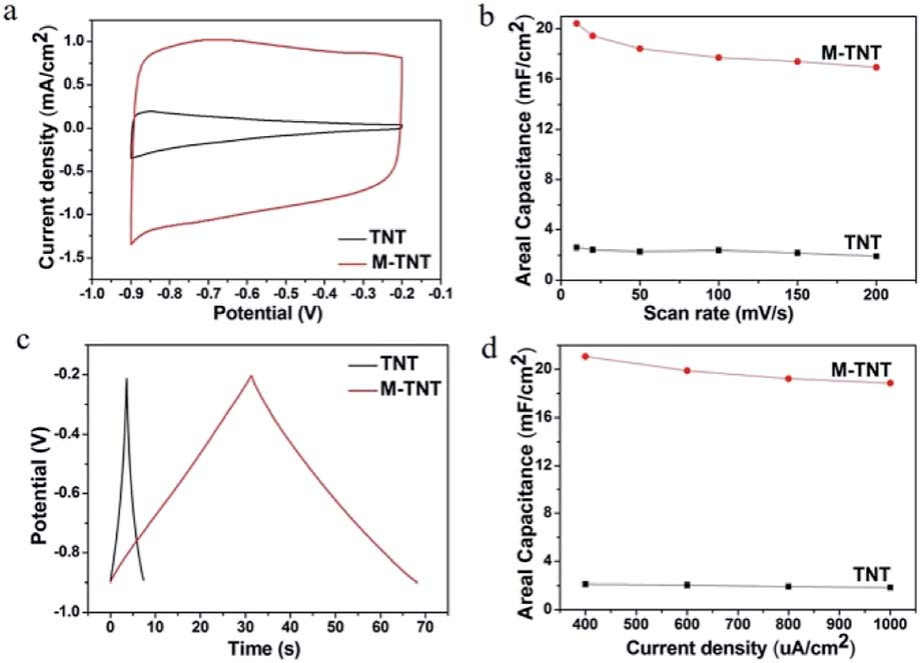
*CV* plots at a scan rate of 50 mV s^−1^ for TNT and M-TNT (a). Areal capacitances of samples measured as a function of scan rate (b). Galvanostatic CD curves at a current density of 0.4 mA cm^−2^ for TNT and M-TNT (c). Areal capacitances obtained at different current densities (d).

To evaluate the electrochemical property of the MoO_3−*x*_/TiO_2_ nanocomposites, electrochemical measurements were performed with a three-electrode system in 1 M Na_2_SO_4_ electrolyte. For a comparison, the pristine TNTs were also intensively studied. Fig. 3a shows the *CV* curves for TNT and M-TNT samples collected at a scan rate of 50 mV s^−1^. The curve of TNT shows a strong dependence of current density on the applied potential. The decrease of current densities at higher potentials results in a sloping profile for TNT. Additionally, a larger area under *CV* curve and higher current response for M-TNT electrode indicate a markedly enhanced electrochemical performance after incorporating with MoO_3−*x*_. The shapes of these *CV* curves remain unchanged as the scan rates increase from 10 to 200 mV s^−1^, indicating good capacitive behavior and high-rate capability of M-TNT (Fig. S1a, ESI†). Fig. 3b shows the calculated areal capacitance of these electrodes as a function of scan rate. The areal capacitances of TNT and M-TNT are 2.26 mF cm^−2^ and 18.40 mF cm^−2^ at a scan rate of 50 mV s^−1^, respectively. Moreover, the sample M-TNT shows good rate capacitance. The specific capacitance of M-TNT drops from 20.44 to 16.92 mF cm^−2^ with a good retention of ∼82.78% of the initial capacitance with scan rates from 10 to 200 mV s^−1^. In contrast, the TNT sample retains only ∼74.0% of the initial capacitance. The enhanced rate capability is attributed to improved electrical conductivity of M-TNT. For all of the samples, the specific capacitance decreases with increasing scan rate, which is usually due to the limitation of charge accumulation at high scan rates.^43^

As reported in literatures, the electrochemical performance of some transition metal oxides can be improved by introducing oxygen deficiency and oxygen non-stoichiometry *via* annealing in inert or hydrogen atmosphere.^20,41,42,45,46^ In our study, the generation of Mo^IV^ oxides and Mo^V^ oxides also can be ascribed to the oxygen depletion inside MoO_3_ network. In comparison with pristine TNTs, the enhanced capacitive performance of M-TNT is attributed to two major improvements upon incorporating with MoO_3−*x*_. First, the conductive MoO_2_ decreases the internal resistance of MoO_3−*x*_/TiO_2_ composite and enables fast charge flow to meet high-rate charge and discharge.^47^ Additionally, oxygen vacancies act as shallow donors and thereby increase the carrier concentration, hence improving electrical conductivity of electrode material.^20,34,36,45^ Second, a Na-intercalating MoO_3_ phase is favorable of intercalation and deintercalation of electrolyte ions, and thus enhancing electrochemical behavior.^26,48^

The electrochemical performance of M-TNT was further studied by galvanostatic CD tests, which were performed in the potential range from −0.9 to −0.2 V. Fig. 3c shows the charge/discharge curves of different electrodes collected at a current density of 0.4 mA cm^−2^. It is to be noted that the discharge time of M-TNT is longer than that of TNT at the same current density. The charge/discharge curves of M-TNT and TNT at different current densities are shown in Fig. S2.†Fig. 3d shows the specific capacitance obtained from galvanostatic CD test at various current densities. The M-TNT shows a remarkable areal capacitance of 21.08 mF cm^−2^ at a current density of 0.4 mA cm^−2^ and high-rate capability with capacitance retention of ∼89.42% as the current density increases from 0.4 to 1 mA cm^−2^. Additionally, the M-TNT shows a larger capacitance than that of TNT at each charge/discharge current density, which is consistent with the results obtained in the *CV* study.

**Fig. 4:**
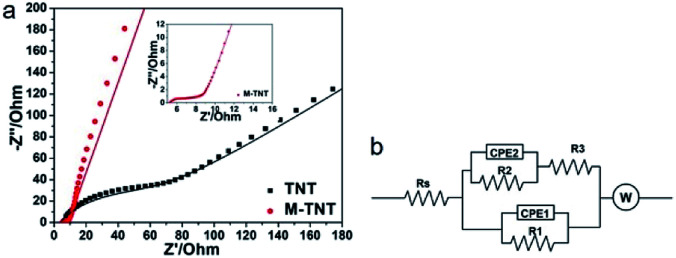
Nyquist plots (a) of TNT and M-TNT with insets showing an enlargement of high-frequency regions. The scattered dots and solid lines are the experimental and fit impedance data, respectively. The corresponding EIS equivalent circuit (b).

EIS measurements were performed to study charge transfer and ions diffusion characteristic of prepared electrode materials. Fig. 4a shows typical Nyquist plots of TNT and M-TNT hybrid. The Nyquist plot of M-TNT exhibits a much lower impedance value than that of TNT. Fig. 4b shows the corresponding equivalent circuit of Nyquist plots. *R*_s_ is the bulk solution resistance and *R*_3_ is the charge–discharge resistance through the nanotubes; *R*_1_ and CPE_1_ are used to simulate the charge-transfer resistances and capacitances of the near-top regions of nanotubes, respectively. *R*_2_ and CPE_2_ values for the near-bottom regions of nanotubes, respectively.^22^ CPE-P is the constant phase element exponent. CPE-T is the capacitance when CPE-P = 1.^49^ W is defined by three following values. W-R is the diffusion resistance, W-T is the diffusion time constant, W-P is a fractional exponent, which has a value near 0.5 with regard to the finite length diffusion.^50^Table 1 lists the fitted values for the elements of TNT and M-TNT. As for *R*_2_ and *R*_3_, the calculated values show no important variation before and after coated with MoO_3−*x*_, which is attributed to the unchanged structure of TiO_2_ as observed from XRD patterns and XPS spectra. Notably, *R*_1_ is obviously decreased from 110 Ω for TNT to 2.5 Ω for M-TNT, which is resulted from the additional deposition of MoO_3−*x*_ layer on the surface of TiO_2_ nanotubes. The value of CPE_1_-T remains virtually the same in the two electrodes, suggesting a double layer capacitance.^51^ From Fig. S3,† the phase angle deviating from a perfect 90° confirms the existence of pseudocapacitance.^45^ Additionally, the small value of Warburg diffusion impedance indicates fast ion diffusion.^50^ The existence of conductive MoO_2_ and oxygen vacancies from MoO_3−*x*_ enhances electrical conductivity, thus improving kinetics of ions transport in the electrode.

**Table 1:** Equivalent circuit parameters for TNT and M-TNT

Equivalent circuit elements	Fitting values	
	TNT	M-TNT
*R*_s_ (Ω)	3	5.1
*R*_1_ (Ω)	110	2.5
CPE_1_-T	7.352 × 10^−6^	2.399 × 10^−6^
CPE_1_-P	0.752	0.696
*R*_2_ (Ω)	110	110
CPE_2_-T	0.002	0.080
CPE_2_-P	0.141	0.145
*R*_3_ (Ω)	5	4.0
W-R	1031	5.197
W-T	0.119	0.020
W-P	0.479	0.426

**Fig. 5:**
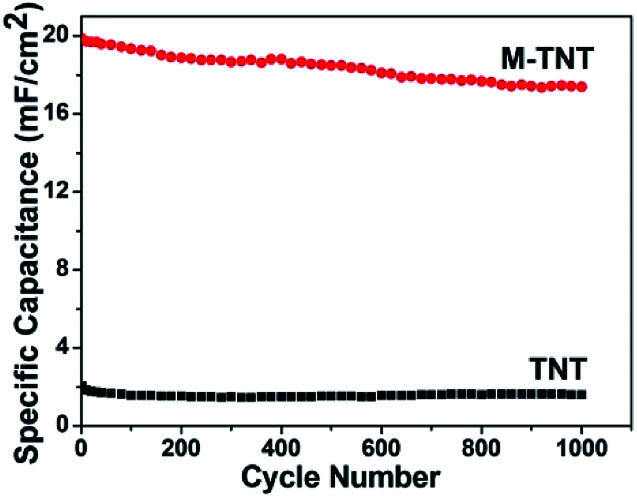
Cycle performance of TNT and M-TNT measured at a current density of 0.6 mA cm^−2^ up to 1000 cycles.

The cyclic charge–discharge measurements were performed to evaluate the electrochemical durability of prepared electrodes. As shown in Fig. 5, the capacitance retentions for TNT and M-TNT are about 78.5% and 87.4% after 1000 cycles, respectively. Thereby, the higher specific capacitance and better cycling stability indicate that the MoO_3−*x*_/TiO_2_ is a promising electrode material.

**Fig. 6:**
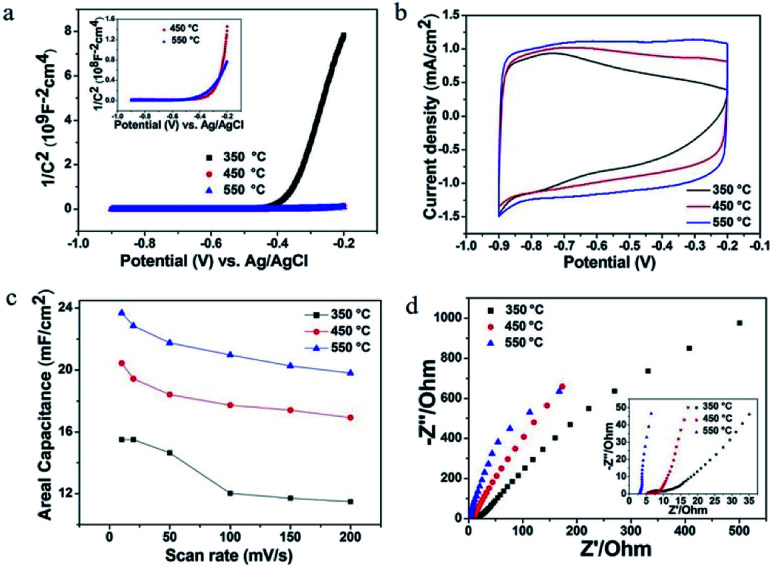
Mott–Schottky plots for samples at different annealing temperatures (a). *CV* plots at a scan rate of 50 mV s^−1^ (b), areal capacitances obtained as a function of scan rate (c) and Nyquist plots (d) for deposited samples annealed at different temperatures.

**Fig. 7:**
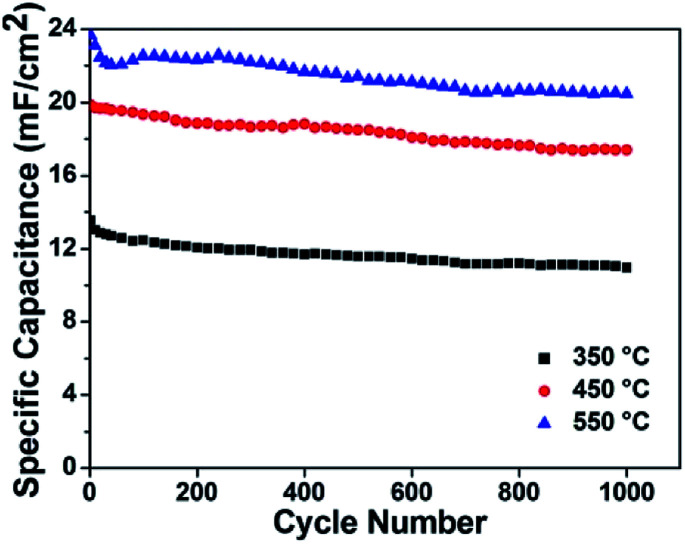
Cycle performance of these samples measured at a current density of 0.6 mA cm^−2^ up to 1000 cycles.

The above-mentioned experimental results confirm the contribution of MoO_3−*x*_ within composite electrode materials. As the formation of substoichiometric oxides is sensitive to thermal conditions, it motivated us to study the effect of annealing temperatures on the structure and electrochemical performance of MoO_3−*x*_/TiO_2_ hybrids. From XRD patterns (Fig. S4†), we discover peaks of crystalline MoO_3−*x*_ tend to appear at higher temperatures (*i.e.* 450 °C and 550 °C). Compared with the sample annealed at 450 °C, the slight shift of MoO_3−*x*_ peaks for the sample annealed at 550 °C is attributed to a change of lattice constant due to the oxygen vacancies.^42^ Additionally, the formation of the thermodynamically stable phase of TiO_2_ (*i.e.* rutile) indicates transformation of anatase to rutile when annealed at 550 °C. Fig. S5† shows SEM images of deposited samples annealed at 350 °C and 550 °C. According to XPS analyses (Fig. S6 and Table S1†), higher amount of Mo^5+^ and Mo^4+^ for sample annealed at 550 °C suggests a larger concentration of oxygen vacancies. In order to obtain a better understanding of the effect of annealing temperatures on electrical properties, Mott–Schottky plots were drawn based on capacitances derived from the imaginary part of the impedance at each potential with 1 kHz frequency. The calculated carrier densities of deposited samples annealed at 350 °C, 450 °C and 550 °C are 7.14 × 10^17^ cm^−3^, 3.12 × 10^19^ cm^−3^, 7.31 × 10^19^ cm^−3^, respectively (Fig. 6a). Mott–Schottky studies reveal 2 orders of magnitudes enhancement of carrier density when the annealing temperature increases from 350 to 550 °C, which is possibly due to the improved crystallinity of MoO_3−*x*_ as well as the creation of oxygen vacancy states. Fig. 6b and c shows *CV* plots at a scan rate of 50 mV s^−1^ and areal capacitances obtained as a function of scan rate, respectively. As expected, the deposited sample annealed at 550 °C yields the highest areal capacitance of 23.69 mF cm^−2^ at a scan rate of 10 mV s^−1^. The EIS analysis indicates that the sample annealed at 550 °C has good charge transfer performance and ion diffusion capability (Fig. 6d). Fig. S7† shows the galvanostatic CD curves for samples annealed at different temperatures and areal capacitances obtained at different current densities. The areal capacitances for deposited samples annealed at 350 °C and 550 °C are 14.57 mF cm^−2^ and 24.74 mF cm^−2^ at a current density of 0.4 mA cm^−2^, respectively. We also performed cycling measurements to examine the long-term stability of these MoO_3−*x*_/TiO_2_ nanotube composites (Fig. 7). The capacitance retention of composite electrodes prepared at 350 °C and 550 °C are 80.9% and 86.6%, respectively. From ESI (Table S1†), we can observe the existence of MoO_3_ for all samples. Our previous study discovered that detachment of MoO_3_ and structure change led to the rapid capacity decay (∼12%) in the initial cycles.^26^ Although oxygen vacancies are capable to stabilize the crystalline structure during cycling, the suitable concentration of oxygen deficiency plays an important role in achieving good cycling performance.^36,40^ Hence, the sample prepared at 450 °C exhibits better cycling stability than those of others. Additionally, a fluctuation in the specific capacitance during cycling is possibly due to “activation process”^52,53^ and the uneven electrolyte wetting of the phases of MoO_2_ and MoO_3_.^40^

## 4. Conclusion

In our study, MoO_3−*x*_/TiO_2_ nanotube composites were synthesized by galvanostatic depositions and then annealed in argon atmosphere. Electrochemical measurements confirm the contribution of MoO_3−*x*_ and enhancement in capacitive performance within composite electrode materials. We also have demonstrated that the electrochemical properties of MoO_3−*x*_/TiO_2_ nanotube composites can be enhanced by controlling annealing temperatures. The electrode prepared at 550 °C yields highest specific capacitance of 24.74 mF cm^−2^ at a current density of 0.4 mA cm^−2^ and good capacitance retention (86.6%) even after 1000 continuous charge–discharge cycles in Na_2_SO_4_ electrolyte. The ease of synthesis and exceptional electrochemical properties suggest MoO_3−*x*_/TiO_2_ nanotube is a promising supercapacitor electrode material.

## Conflicts of interest

There are no conflicts to declare.

## Supplementary Material

RA-008-C8RA02744G-s001
