# Supplementary material for: MoO3−x-deposited TiO2 nanotubes for stable and high-capacitance supercapacitor electrodes
Source: RSC Adv. 2018 Jun 13;8(39):21823–8. doi: 10.1039/c8ra02744g (PMC9081865; doi:10.1039/c8ra02744g)
Supplement: RA-008-C8RA02744G-s001 [file RA-008-C8RA02744G-s001.pdf]

Electronic Supplementary Material (ESI) for RSC Advances.
This journal is © The Royal Society of Chemistry 2018

Supporting information

MoO_{3-x}-deposited TiO_2 nanotube for stable and high-capacitance supercapacitor electrodes

Shupei Sun, Yu Sun, Jiang Wen, Bo Zhang, Xiaoming Liao*, Guangfu Yin, Zhongbing Huang, Ximing Pu

College of Materials Science and Engineering, Sichuan University, Chengdu, Sichuan 610065, China

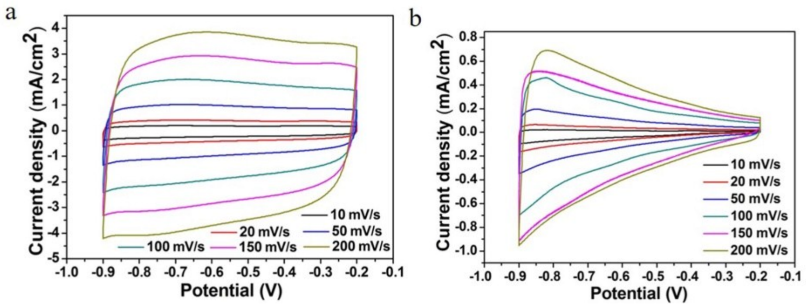

Fig. S1 The CV plots measured at different scan rates for M-TNT (a) and TNT(b).

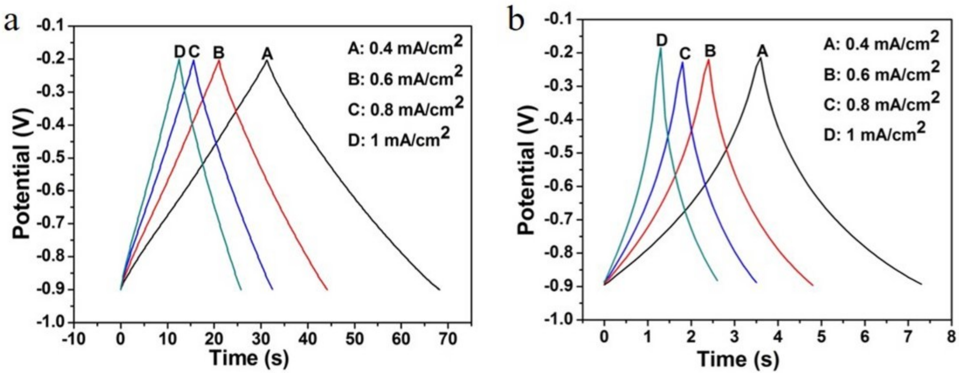

Fig. S2 Galvanostatic charge-discharge curves for M-TNT (a) and TNT (b) achieved from 0.4 mA cm^{-2} to 1 mA cm^{-2}.

* Corresponding author. Fax: +86 28 85413003. E-mail: sherman_xm@163.com (X.M. Liao).

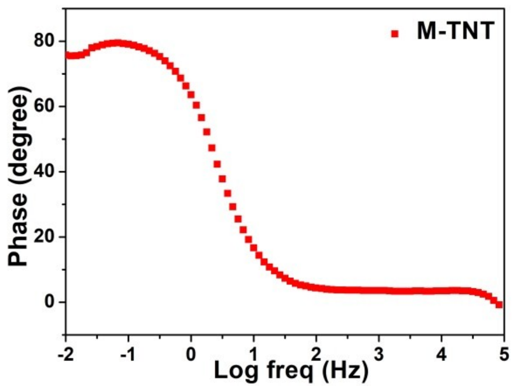

Fig. S3 Bode plot of M-TNT.

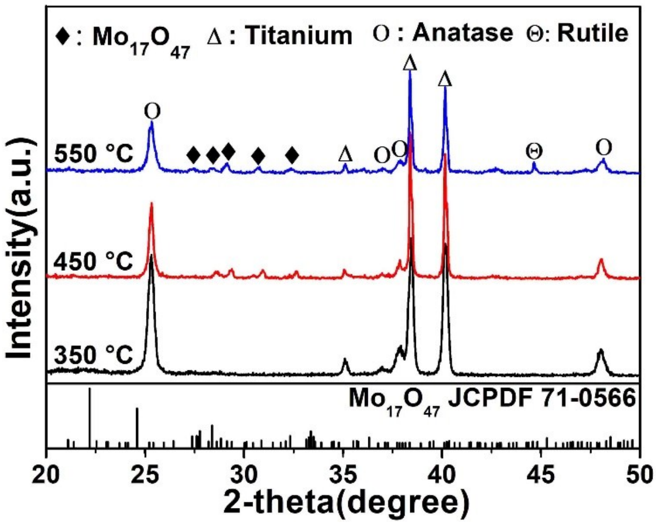

Fig. S4 XRD patterns of deposited samples annealed at different temperatures.

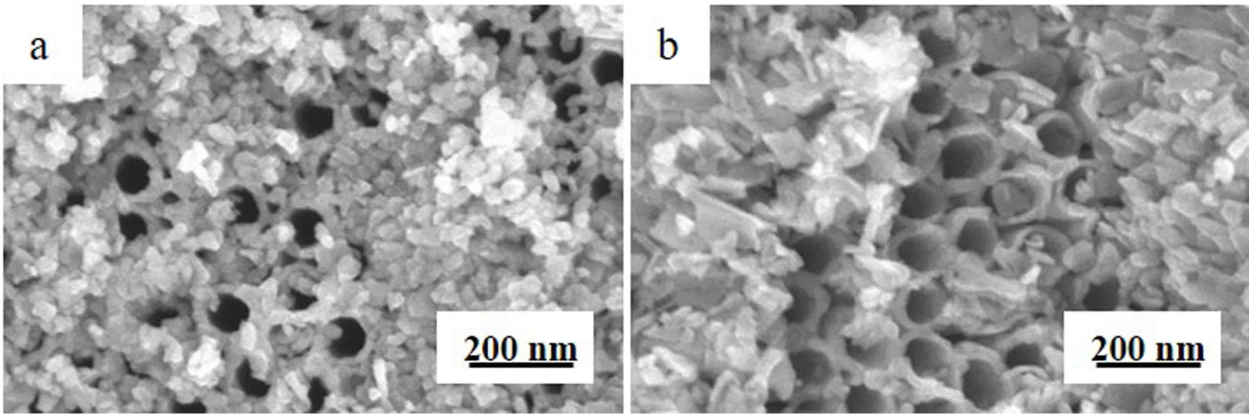

Fig. S5 SEM images of deposited samples annealed at 350 ˚C (a) and 550 ˚C (b).

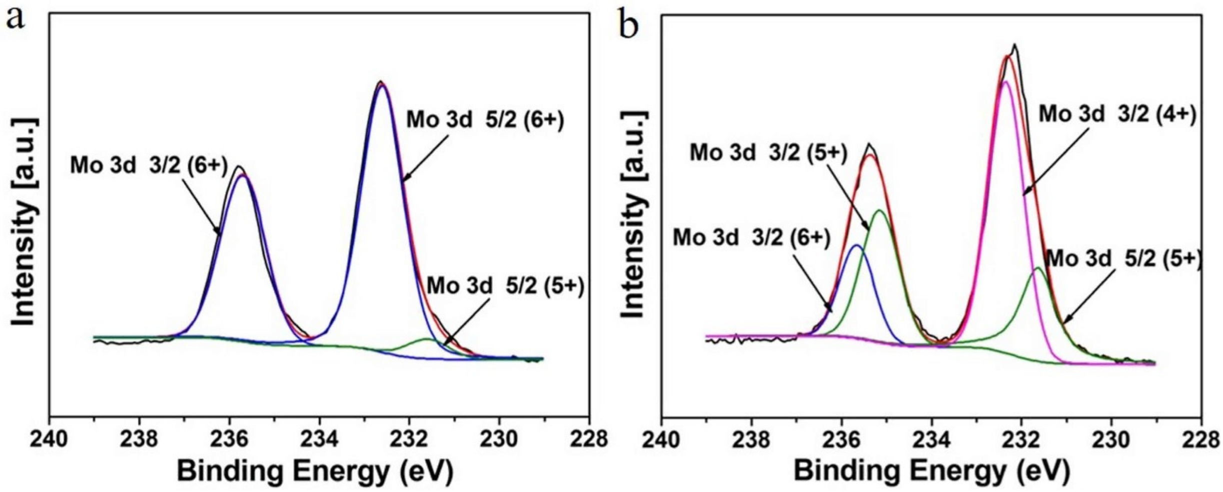

Fig. S6 XPS spectra in the Mo 3d binding energy region of deposited samples annealed at 350 ˚C (a) and 550 ˚C (b).

Table S1 Relative content (at %) of Mo^{6+}, Mo^{5+} and Mo^{4+} within deposited samples annealed at different temperatures.

	350 ˚C	450˚C	550˚C
Mo^{6+}	95.97	59.74	14.35
Mo^{5+}	4.03	28.12	42.10
Mo^{4+}	-	12.14	43.55

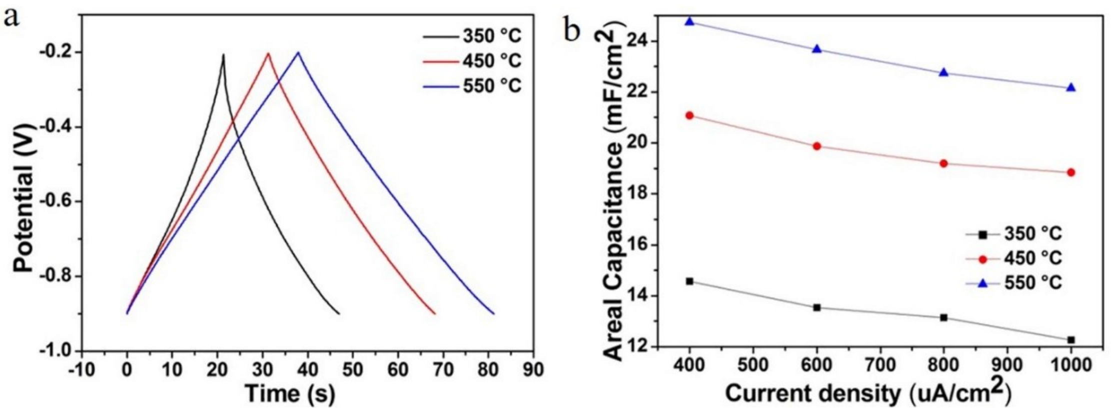

Fig. S7 Galvanostatic CD curves measured at a current density of 0.4 mA cm^{-2} (a), and areal capacitances of these samples obtained at different current densities (b).
